# Structural Insights into the Dehydration and Rehydration of Gypsum

**DOI:** 10.1021/acs.cgd.4c01414

**Published:** 2025-04-24

**Authors:** Miguel Burgos-Ruiz, Kerstin Elert, Alejandro B. Rodriguez-Navarro, Encarnacion Ruiz-Agudo, Carlos Rodriguez-Navarro

**Affiliations:** † Department of Mineralogy and Petrology, Faculty of Sciences, 16741 University of Granada, Avenida Fuentenueva S/N, Granada 18002, Spain; ‡ Escuela de Estudios Árabes, CSIC, Cuesta Del Chapiz, Granada 18010, Spain

## Abstract

The formation and transformation of phases within the CaSO_4_–H_2_O system are crucial in natural and industrial processes. Despite intensive research, however, the mechanisms of gypsum’s (CaSO_4_·2H_2_O) thermal dehydration and rehydration remain unclear. Here we investigated the crystallographic and textural relationship between parent and product phases during the dehydration and rehydration of gypsum single crystals using two-dimensional X-ray diffraction (2D-XRD), field emission scanning electron microscopy (FESEM), in situ and ex situ transmission electron microscopy (TEM), and selected area electron diffraction (SAED). Our findings reveal that the dehydration products, bassanite (CaSO_4_·0.5H_2_O) or soluble anhydrite (γ-CaSO_4_), exhibit no clear structural relationship with the parent phase in the case of millimeter-sized gypsum crystals. Conversely, a topotactic replacement reaction is observed for micrometer-sized gypsum crystals. This disparity is due to localized nonoriented crystallization of α-hemihydrate in microhydrothermal cavities within large crystals resulting from local gypsum dehydration, whereas in smaller gypsum crystals unimpeded H_2_O release results in oriented β-hemihydrate via a solid-state mechanism. Rehydration proceeds via a dissolution–precipitation mechanism, without structural continuity between parent and product phases. Importantly, gypsum relicts in partially dehydrated pseudomorphs serve as template for the epitaxial crystallization of gypsum during rehydration. These findings provide valuable insights for optimizing hemihydrate production and hydraulic setting, benefiting both construction and biomedical applications.

## Graphical abstract

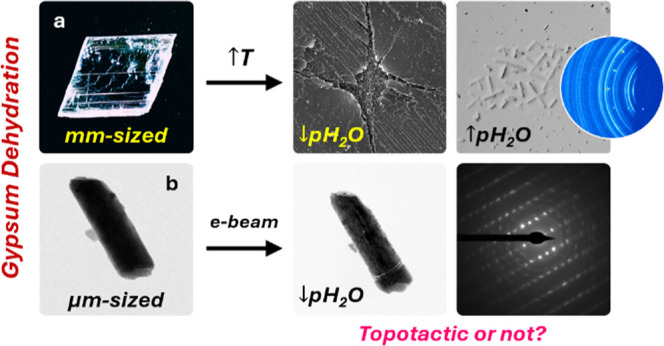

## 1 Introduction

Gypsum (CaSO_4_·2H_2_O) is the most abundant sulfate salt on Earth and is considered as one of the most important industrial minerals in the world. The formation of gypsum and the phase transitions that occur in the CaSO_4_–H_2_O system have thus been extensively investigated for more than 100 years. Thermal processing of gypsum (calcination at <180 °C) leads to its dehydration, producing bassanite (hemihydrate, CaSO_4_·0.5H_2_O) and/or soluble anhydrite (γ-CaSO_4_). These are the main components of plaster of Paris, a mineral binder used since prehistoric times as structural and decorative building materials, including stuccoes and fire-resistant wall coatings and ceilings.^1^ Calcium sulfate phases are important not only in construction but also in soil amendment and water retention in horticulture, as fillers in the paper and pharmaceutical industries, and for bone and dental grafting, among other technical applications.^2–4^ The dehydration and (re)­hydration reactions of the “gypsum cycle”, analogous to the “lime cycle”,^1^ have been extensively studied. These reactions are key to understanding the characteristics and use of plaster of Paris as a binder and the geological (terrestrial or extraterrestrial) events that led to the formation of calcium sulfate minerals and their phase transitions. In this respect, gypsum dehydration has been long studied as a model for seismogenic and metamorphic dehydration processes that occur in Nature (e.g., during plate subduction), where the release and flux of water has significant sedimentary and tectonic implications.^5–7^

There is ample evidence showing the tight interdependence between temperature and water vapor partial pressure (*p*H_2_O) conditions and the products obtained after gypsum calcination. While at low *p*H_2_O γ-CaSO_4_ is obtained in a single step, the dehydration of gypsum follows a two-step mechanism under moderate-to-high *p*H_2_O involving the formation of bassanite as intermediate phase.^8–14^ The dehydration of gypsum can thus be described by the following set of reactions

1 $${CaSO}_{4}{\cdot 2H}_{2}O\left(s\right)\to {CaSO}_{4}{\cdot 0.5H}_{2}O\left(s\right)+{1.5H}_{2}O\left(g\right)$$

2 $${CaSO}_{4}{\cdot 0.5H}_{2}O\left(s\right)\to {\gamma -CaSO}_{4}\left(s\right)+{0.5H}_{2}O\left(g\right)$$

3 $${CaSO}_{4}{\cdot 2H}_{2}O\left(s\right)\to {\gamma -CaSO}_{4}\left(s\right)+{2H}_{2}O\left(g\right)$$

Furthermore, γ-anhydrite transforms into β-anhydrite (“insoluble anhydrite”) at ∼280 °C under normal atmospheric pressure conditions. This phase is more stable and less soluble than gypsum and bassanite at temperatures higher than ∼42 °C in aqueous environments.^15^ Insoluble anhydrite can also be obtained directly from gypsum under extremely low *p*H_2_O conditions (e.g., high vacuum) or in high salinity hydrothermal environments.^16,17^

Additionally, the thermal dehydration of gypsum has been reported to involve the formation of subhydrates with formula CaSO_4_·*n*H_2_O (0 < *n* < 0.8), although the existence of such subhydrates has been challenged.^18^ Indeed, there is still controversy regarding this aspect and even new phases, such as “hydro-γ-anhydrite”, have been theorized to form during the full gypsum dehydration pathway.^19^

Despite the difference in structural water content among the CaSO_4_·*n*H_2_O phases (i.e., 0 < *n* < 2), phase transitions in the CaSO_4_–H_2_O system below 280 °C take place with little structural alterations, being commonly considered topotactic.^11,20,21^

Gypsum crystallizes in the monoclinic system. Note, however, that different unit cell choices and space groups (e.g., *A*2/a; *C*2/c, *I*2/a, and *I*2/c) have been reported.^22–27^ Here we have adopted the space group *I*2/a,^25^ also used by Beaugnon et al.^28^ in a recent study on the structural features of the thermal dehydration of gypsum single crystals.

Bassanite shows two polymorphs, α and β, whose structures have been respectively described as monoclinic (space group *I*2) and trigonal (space group *P*3_2_21),^29^ although a more recent crystal structure refinement shows also a monoclinic structure for the β-polymorph (space group *I*2),^30^ with contact twinning accounting for the apparently trigonal structure.^18^ In essence, these two structures are identical, and they exhibit channels along the *c*-axis (diameter of 6.93, 7.18 and 6.71 Å, as measured for the S··S, Ca1··Ca1 and Ca2··Ca2 distances, respectively), which allows to easily accommodate or release weakly bound water molecules.

Soluble anhydrite crystallizes in the hexagonal system (space group *P*6_2_22), although an orthorhombic structure has been reported (space group *C*2*mm*) in which the channels along the *c*-axis direction (diameter of 6.70 Å) are preserved after the removal of water from bassanite.^31^ In fact, the structure of soluble anhydrite is typically understood as a dehydrated and slightly more compact version of the hemihydrate phases. Finally, orthorhombic insoluble β-anhydrite (space group *Amma*)^32^ shows a more compact structure than the other phases but preserves the same strong Ca–SO_4_ chains along [001] and the structural channels of soluble anhydrite.

The similarities between these phases explain why gypsum dehydration (whether in one or two steps) and the transformation of soluble into insoluble anhydrite are often described as topotactic and pseudomorphic processes, assuming that atomic arrangement and crystal morphology are preserved after the release of structural water molecules.^20^ However, X-ray diffraction analyses have not confirmed a clear structural relationship between parent and product phases during the thermal decomposition of gypsum, raising doubts about the topotactic nature of such a phase transformation.^11,20^ In contrast, TEM-SAED analyses suggest that the transformation is indeed topotactic,^20^ which would explain the observed preferred orientation of fractures and dehydration products.^20,28^ Considering the structural similarities between gypsum and hemihydrate, as well as soluble anhydrite phases, it could be argued that the hydration of the latter could also be topotactic. However, most evidence reported so far concludes that the hydration of bassanite (or soluble anhydrite, which rapidly hydrates in air to form bassanite) is a dissolution–precipitation process.^33,34^ Nonetheless, no detailed crystallographic study has been performed so far to disclose whether the hydration step is structurally controlled.

These phase transitions, and whether they are structurally controlled or not, are crucial to predict the properties of the resulting product phase(s).^35^ However, the textural and crystallographic relationships between the CaSO_4_·*n*H_2_O phase(s) involved in the calcination of gypsum and the hydration of bassanite (and soluble anhydrite) are still not properly understood.

Here we explore the micro- and nanostructural changes that occur during the calcination of gypsum single crystals under low *p*H_2_O conditions and the subsequent vapor phase hydration of bassanite by means of two-dimensional X-ray diffraction (2D-XRD), field emission scanning electron microscopy (FESEM), in situ and ex situ transmission electron microscopy (TEM), and selected area electron diffraction (SAED) analyses. Our study shows that the solid-state decomposition of gypsum strongly depends on the crystal size. The macroscopic ex situ analysis results show that these reversible processes produce crystals with no apparent shared crystallographic relationships to the parent single gypsum crystal (during dehydration) or the final dehydrated pseudomorph (during rehydration). However, the nano- and microscopic results reveal a clear structural relationship between parent and product phases during dehydration. Further insights into these discrepancies are discussed here.

## 2 Experimental Section

### 2.1 Calcination and Rehydration of Gypsum

For the analysis of the thermal decomposition of gypsum, centimeter-size optical quality (transparent and free from inclusions) gypsum crystals (from Naica, Mexico) were cleaved along (010) using a scalpel to obtain millimeter-sized (approximately 2 × 2 × 1 mm) single crystals. The purity of the gypsum crystal was demonstrated by energy dispersive X-ray fluorescence spectroscopy (EDS, coupled to an SEM). Multiple EDS maps and point analyses on the Naica gypsum crystals showed the presence of Ca, S, and O, with no traces of any other element (Supporting Information, Figure S1). Three different sets of gypsum crystals were separately heated in an air-ventilated oven, from 25 °C to 100, 130, and 150 °C at a heating rate of 5 °C min^–1^. Subsequently, samples were annealed at the target temperature for 30 min. Heating at 100 °C resulted in a weight loss of 2.2 ± 0.5%. Considering that the loss of two water molecules from complete gypsum dehydration corresponds to a weight loss of 20.91%, the percentage of conversion of gypsum was 10.5% (corresponding to 14.3% conversion of gypsum into hemihydrate). For the run at 130 °C the weight loss was 13.1 ± 3.3%, which corresponds to an average conversion of 62.6% (corresponding to 83.5% conversion of gypsum into hemihydrate). Finally, in the run at 150 °C, a weight loss of 20.5 ± 1.4% was observed, which corresponds to ∼100% conversion (in this case, involving the formation of soluble anhydrite as end-product). The resulting partially or fully dehydrated gypsum pseudomorphs (i.e., product preserving the overall shape of the parent crystals) were collected and carefully stored in closed glass vials under dry N_2_ atmosphere until further analysis.

Dehydrated gypsum pseudomorphs were subjected to rehydration. The specimens were placed in a sealed container for 12 months at 25 °C and 100% relative humidity. We chose to hydrate the pseudomorphs in high relative humidity conditions to avoid sample disintegration by direct contact with liquid water (see Supporting Information, Figure S2). The latter precluded the analysis of the structural/crystallographic relationship between parent and product phases during the rehydration process. Note that while the mode of hydration (i.e., water vapor vs liquid water) affects the kinetics and the textural features of product gypsum,^36,37^ they do not affect the hydration mechanism, which in both cases is a dissolution–precipitation mechanism.^38^

### 2.2 Mineralogical and Textural Analysis of Samples

Optical microscopy (OM) photomicrographs of {010} cleavage surfaces of dehydrated gypsum pseudomorphs were acquired using a Leica DVM2000 digital microscope and a Zeiss Jenapol U (using polarized light in transmission).

Textural and EDS analysis of {010} cleavage surfaces of carbon-coated gypsum single crystals and calcined pseudomorphs (before and after rehydration) were carried out by means of FESEM, using a Carl Zeiss Auriga (10^–6^ Pa vacuum, 3 kV acceleration voltage, secondary electron imaging mode) and a SEM Phenom XL (10^–1^ Pa vacuum, 15 kV acceleration voltage and backscattered electron image mode). The 2D-XRD analysis of gypsum single crystals, their low temperature pseudomorphs, and the rehydration products, was performed using a Bruker D8 Venture X-ray single-crystal diffractometer equipped with a PHOTON area detector. Patterns were collected using a 0.2 mm collimated beam, 120 s exposure time and Mo–Kα radiation (λ = 0.7107 Å). Samples were measured in reflection mode (2θ = 20 and ω = 10°), and a sequence of 2D diffraction patterns were collected during a Phi scan with 5° steps (72 frames in total). The XRD2Dscan v.8.0 (Malvern PANalytical, The Netherlands) software package was used to analyze the collected 2D-XRD patterns and to prepare pole figures showing the crystallographic/structural relationship between parent and product phases.

To evaluate the effect of crystal size on the thermal dehydration of gypsum and rehydration of pseudomorphs, crystallographic features of gypsum and bassanite microcrystals (obtained by thermal treatment of powder gypsum, see below) and partially hydrated bassanite particles were determined by means of TEM, using a Thermo Fisher Scientific TALOS F200X microscope (operated at 200 kV acceleration voltage). Gypsum powder samples were prepared by grinding optical quality gypsum crystals (Naica, Mexico) using an agate mortar. A fraction of the obtained powder was subjected to thermal dehydration (1 h at 130 °C) to obtain microcrystalline bassanite samples. The resulting powder samples were then sieved below 35 μm, dispersed in absolute ethanol and sonicated for 20 s before being deposited on carbon-coated Cu grids. The prepared samples were plasma cleaned, and TEM micrographs were obtained using a 40 μm objective aperture.

In situ TEM was employed to accurately describe the electron beam induced dehydration of gypsum and bassanite at the nanoscale. During this process, sequential low- and high-resolution micrographs were obtained. In the case of low-resolution imaging, the e-beam was focused on a small area of the gypsum or bassanite crystal for 90–360 s and sequential micrographs were collected (before and after e-beam induced dehydration). Similarly, dehydration was observed during high-resolution imaging (typically dehydration occurred after 10–30 s e-beam exposure). In all cases the electron dose rate or fluence (*F*) was calculated using the following equation:

4 $$F=\frac{I}{eA}$$

where *I* is the e-beam intensity, which ranged between 4.9 and 5.4 nA, e is the electron charge (1.602 × 10^–19^ C) and A is the e-beam illuminated area (Å^2^). The total electron dose was calculated by multiplying the fluence per total exposure time (in s).

Finally, detailed crystallographic information was obtained by measuring SAED patterns collected using a 10 μm objective aperture (∼400 nm diameter illuminated area) and fast Fourier transform (FFT) patterns of areas imaged at high resolution (HRTEM).

## 3 Results and Discussion

### 3.1 Dehydration of Gypsum

Optical microscopy reveals that gypsum single crystals develop a milky appearance as calcination *T* increases (Figure 1a–d). This change results from the nucleation of thermal pits and extensive fracturing along specific crystallographic directions on the (010) face. High-magnification imaging (Figure 1d,e) shows the formation of numerous hourglass-shaped dehydration nucleation sites (i.e., thermal pits), which typically align with surface defects or discontinuities (e.g., cleavage planes and macro steps, as well as subgrain boundaries). Such structures, characteristic of the thermal dehydration of gypsum single crystals,^28,39,40^ display prominent striation along the bisector of the acute angle formed by two intersecting fractures. The pseudomorphs obtained at 100 °C display some transparent areas (unlike the pseudomorphs heated at higher *T*, which are opaque), thereby allowing OM observation using transmitted light. This enabled us to disclose the textural features of the interior of the partially dehydrated gypsum single crystals. Vacuoles with a meandering contour are observed in the interior of such pseudomorphs (Figure 1g). They formed during the initial dehydration of gypsum because released H_2_O got trapped within the crystal. In these interior areas aggregates of randomly oriented micrometer-sized crystals, in some cases with a clear fibrous habit, are observed (Figure 1h,i). Altogether, these observations suggest that a new phase, with textural features consistent with α-hemihydrate,^39^ crystallized in the interior of the millimeter-sized gypsum crystals within a solution resulting from the water generated during partial, localized dehydration of gypsum. The water entrapped in the interior of the gypsum crystals (e.g., in the vacuoles) equilibrates with its surrounding solid phase via dissolution. Once saturation is reached, the less soluble (at *T* ≥ 100 °C) hemihydrate phase crystallizes.^41^ This is consistent with previous studies of the thermal dehydration of large (millimeter-sized) gypsum single crystals showing the formation of needle-like α-hemihydrate crystals in their interior via a solution crystallization process.^39,42^

**Figure 1:**
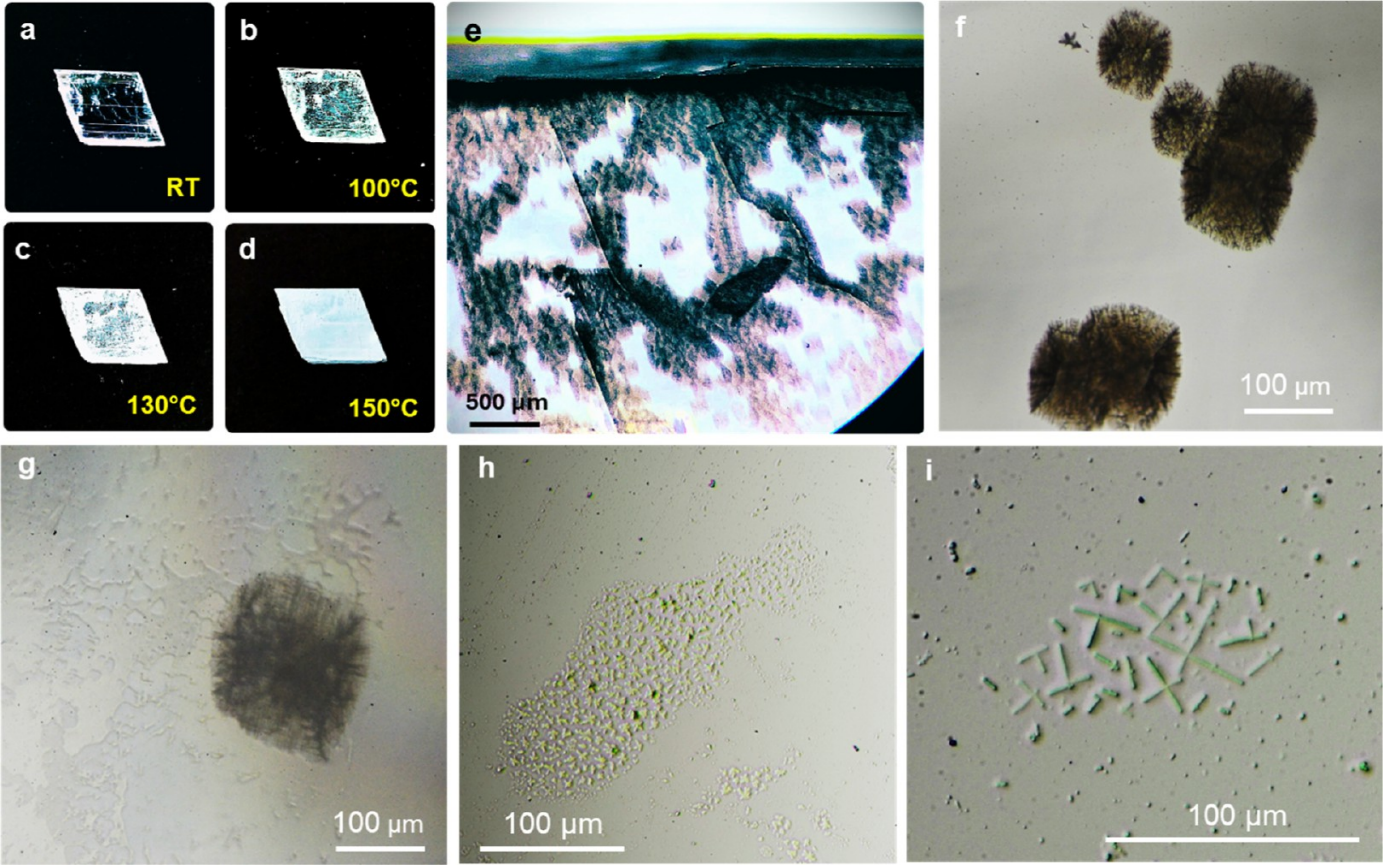
Textural and microtextural variations observed along [010] after thermal dehydration of gypsum single crystals (a,d). The higher magnification optical micrograph (e) shows the nucleation of thermal pits in random positions on the outermost {010} gypsum surfaces; (f) detail of thermal pits on the surface of a gypsum crystal dehydrated at 100 °C; (g) Representative example of vacuoles present in the interior of the partially dehydrated gypsum crystals (underneath a thermal pit); (h) Aggregates of hemihydrate microcrystals nucleated in the interior of the gypsum pseudomorph. (i) Detail of an aggregate of fibrous crystals (α-hemihydrate) formed within the interior of a gypsum crystal partially dehydrated at 100 °C.

The most significant and thoroughly reported dehydration-induced microtextural change observed by FESEM is the formation of cracks (∼0.5–21 μm wide) parallel to the gypsum main cleavage plane (i.e., {010}). This feature reflects the loss of structural water from layers parallel to (010) planes.^20,28,39,43,44^ Water molecules, coordinated to Ca–SO_4_ chains running parallel to [101] within the (010) planes, are removed during dehydration, causing shrinkage along both [010] and [10-1] directions. This results in cracks parallel to the (10-1) planes (or equivalent (-101)) (Figure 2). The release of water molecules (in the form of water vapor) primarily occurs along the cracks parallel to (010), since H_2_O diffusion along [010] is hindered.^45^ However, additional cracks form along [101], [100] and [001] on the (010) surface of dehydrated gypsum (Figure 2a,b), further facilitating H_2_O escape. Hourglass-shaped cracks form on the (010) surface of the pseudomorphs as indicated above (Figure 2a,b). As an additional feature of this type of cracks, nonstriated and bent-up triangular areas are also observed on the obtuse areas of the hourglass structure, which probably form by the combined effect of shear stress release along [101] and compression along [001] (Figure 2b). These textural features of dehydrated gypsum arise after random nucleation of thermal pits on {010} surfaces.^21,28^ The hourglass structures are apparently formed by coalescence of shrinkage cracks and formation of parallel hemihydrate/soluble anhydrite crystals arranged in opposing triangular substructures. In this case the cracks follow the (10-1) or (-101) planes. Importantly, no significant shrinkage occurs along [101], which explains why the hemihydrate fibers align in this direction on the (010) plane of dehydrated gypsum.^28^ In agreement with the cracking rules described by Sipple et al.,^20^ and according to the periodic bond chain (PBC) theory developed by Hartman and Perdok,^46^ the long and continuous cracks along [101] on (010) planes are parallel to the strongly bonded Ca–SO_4_ chains (primary PBC in gypsum; space groups *I*2/*a* and *I*2/*c*), which should remain invariable throughout the atomic rearrangements that occur during the dehydration of gypsum. Considering this, cracks should also form parallel to the other two stable PBCs in gypsum.

**Figure 2:**
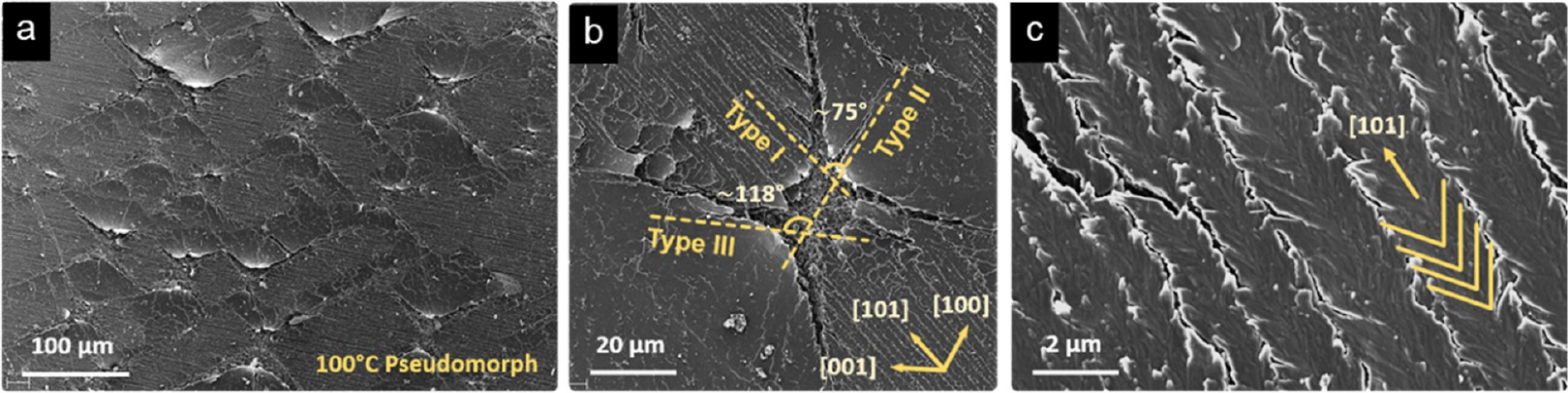
FESEM photomicrographs of gypsum pseudomorphs. (a) General overview of thermal pits on (010)_Gyp_ showing the characteristic hourglass pattern; (b) Detail of a hourglass thermal pit resulting from microfractures oriented along [101], [100] and [001] (i.e., type I, II and III, respectively) formed upon dehydration of gypsum, as defined for the *I*2/a space group; (c) detail of type I microfractures showing the formation of bassanite/soluble anhydrite microneedles adopting a characteristic fishbone pattern (highlighted by the yellow lines).

Although the direction of PBCs depends on the selected space group, two of these strong PBCs follow the [001] and [100] directions, while a third one follows [101], as indicated above. Here, we selected the *I*2/*a* space group to describe the observed phenomenology. In fact, three types of cracks with different sizes (from larger to smaller size, type I, II and III) are visible on (010)_Gyp_. As shown in Figure 2b, type II and III cracks systematically intersect at an angle of ∼118°, which is approximately equal to the β parameter of the monoclinic unit cell of gypsum, while type I and III, and type I and II cracks intersect at an angle of ∼70 and ∼60°, respectively, which are equal to the angles formed by the *a*- and *c*- axes with the [101] direction in the gypsum unit cell (independently of the space group). Therefore, it seems reasonable to assume that the observed hourglass-shaped cracks develop on weakly bonded planes parallel to strong PBCs.

To clarify these crack rules, we compared the polyhedral arrangement in the gypsum structure as observed along [010] for the *C*2/*c*, *I*2/*a* and *I*2/*c* space groups. Figure 3 shows that equivalent PBCs (here denoted as PBC-1, PBC-2 and PBC-3) follow different directions depending on the different spatial descriptors of the gypsum structure and choice of unit cell. To verify the spatial relationships between these three settings, the *C*2/*c* → *I*2/*c* and *C*2/*c* → *I*2/*a* transformation matrices are provided below, allowing the unit cell vectors of the *I*-centered space groups to be described in terms of the vectors of the initial *C*-centered configuration, as follows:

$$C2/c\to I2/c;\left[\begin{array}{l} h_{2}\\ k_{2}\\ l_{2} \end{array}\right]=\left[\begin{array}{lll} -1 & 0 & 0\\ 0 & -1 & 0\\ -1 & 0 & 1 \end{array}\right]\left[\begin{array}{l} h_{1}\\ k_{1}\\ l_{1} \end{array}\right]=\left[\begin{array}{l} {-h}_{1}\\ {-k}_{1}\\ {-h}_{1}+l_{1} \end{array}\right]$$

$$C2/c\to I2/a;\left[\begin{array}{l} h_{2}\\ k_{2}\\ l_{2} \end{array}\right]=\left[\begin{array}{lll} 0 & 0 & -1\\ 0 & 1 & 0\\ 1 & 0 & -1 \end{array}\right]\left[\begin{array}{l} h_{1}\\ k_{1}\\ l_{1} \end{array}\right]=\left[\begin{array}{l} {-l}_{1}\\ k_{1}\\ h_{1}-l_{1} \end{array}\right]$$

**Figure 3:**
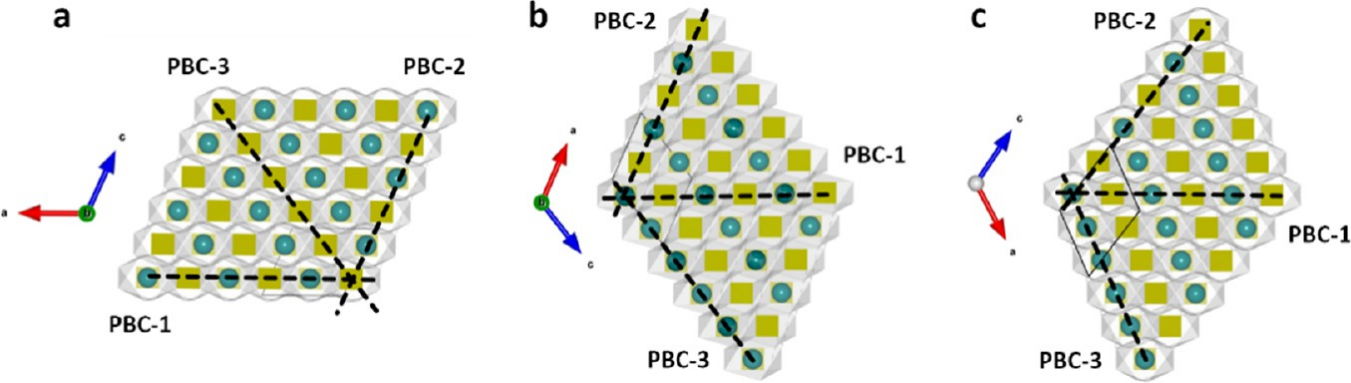
Scheme of the orientation of the main PBCs in the structure of gypsum according to the *C*2/*c* (a), *I*2/*a* (b), and *I*2/*c* (c) space groups.

Consequently, the *I*2/*a* → *I*2/*c* transformation matrix can be also calculated

$$I2/a\to I2/c;\left[\begin{array}{l} h_{2}\\ k_{2}\\ l_{2} \end{array}\right]=\left[\begin{array}{lll} 0 & 0 & -1\\ 0 & -1 & 0\\ -1 & 0 & 0 \end{array}\right]\left[\begin{array}{l} h_{1}\\ k_{1}\\ l_{1} \end{array}\right]=\left[\begin{array}{l} {-l}_{1}\\ {-k}_{1}\\ {-h}_{1} \end{array}\right]$$

Thus, the direction of PBCs and, consequently, crack formation can be defined for the different space groups, as summarized in Figure 3 and Table 1.

**Table 1:** Summary of the Orientation of the Main PBCs in the Structure of Gypsum According to the Relationships between the *C*2/*c*, *I*2/*a* and *I*2/*c* Space Groups^a^

space group	*a*-axis	*b*-axis	*c*-axis		PBC-1	PBC-2	PBC-3
C2/c	[100]	[010]	[001]		[100]	[001]	[101]
I2/a	[1̅00]*	[010]*	[1̅01]*		[101]	[100]	[001]
I2/c	[001̅]*	[01̅0]*	[101̅]*		[101]	[001]	[100]

a Main axial directions corresponding to the *I*-centered unit cells are given as transformed from the *C*2/*c* space group.

From a crystallographic point of view, it is good practice to analyze physical properties and crystallization mechanisms (e.g., crack development, topotactic relationships, epitaxial growth) according to well-defined space group descriptors.^47^

Takahashi and Setoyama,^43^ and Beaugnon et al.^28^ showed that dehydration of gypsum resulted in the formation of cracks intercalated with acicular anhydrite domains in which microneedles were arranged in a regular fishbone pattern. We observe the same pattern of rod-shaped β-bassanite and/or γ-anhydrite microcrystals on {010} gypsum faces, with their long axis oriented at an angle of ∼35–45° with respect to the [101] direction (Figure 2c). Note that if γ-anhydrite is formed, it will rapidly transform into bassanite upon air exposure at room *T*, making it difficult to identify its presence during subsequent analysis. According to Beaugnon et al.,^28^ cracks along [101] result from shrinkage along [10-1], whereas the fishbone structure results from shear stresses associated with the unit cell expansion along [101] following the transition from gypsum into hemihydrate. Such stress energy is released via shear strain, likely along cleavage planes, so that the overall [101] orientation of the fibers is not lost. Yet, this implies multiple bond-breaking along the Ca–SO_4_ chains, a process which seems not thermodynamically favorable. Alternatively, it could be argued that these fishbone structures are the result of multiple mechanical twining along the 101 contact twin law.^48^ Interestingly, these microtextural features form independently of the calcination temperature (i.e., considering the explored *T* range), as the general aspect of cracks and hemihydrate fishbone patterns is similar in all the studied cases.

Altogether, these textural features present on the surface of the pseudomorphs indicate a topotactic solid-state mechanism for gypsum’s thermal dehydration. Note, however, that different textural features might develop in the interior of the gypsum crystals (where water release is hindered) due to cracking and deformation related to the molar volume differences between parent and product phases, and/or secondary nucleation and growth processes.^39,42,49^ The latter is supported by the presence of craters on the (010) surface of dehydrated gypsum, revealing randomly oriented (nano)­crystals beneath (Figure 4a,b), as well as the newly formed, randomly oriented fibrous crystals within the interior of the gypsum single crystals observed with OM (Figure 1h,i).

**Figure 4:**
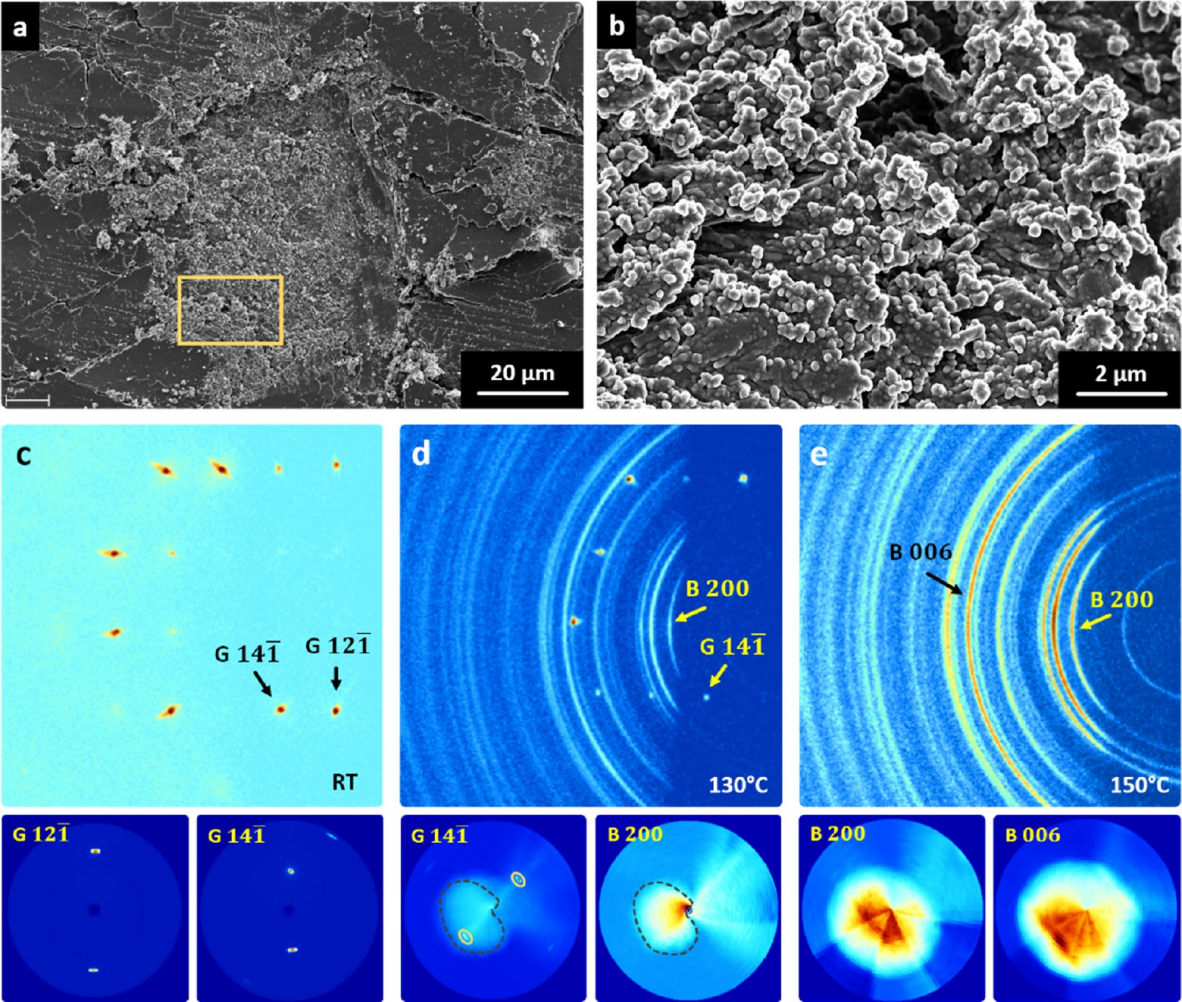
FESEM micrographs showing a micrometric thermal pit (a,b) formed during gypsum single crystal dehydration (the image in (b) corresponds to the rectangular area marked in (a)). The globular nanoparticle texture suggests secondary precipitation of bassanite by dissolution and nucleation processes, driven by high *p*H_2_O and liquid water accumulation beneath the outermost {010} gypsum surfaces. 2D-XRD patterns and pole figures of an unaltered gypsum single crystal (c) and its pseudomorphs (130 °C, (d); 150 °C, (e), reveal bassanite formation without clear preferred orientations (i.e., ring pattern). The calculated pole figures correspond to the Bragg reflections indicated by arrows. Legend: G, gypsum; B, bassanite. Relevant *hkl* spots/rings are indicated.

To disclose whether a preferred orientation existed between parent and product phases, 2D-XRD analyses of the gypsum pseudomorphs were performed using Mo–Kα radiation, which is ∼10 times more penetrative than standard Cu–Kα, so that the interior of the pseudomorphs could be analyzed. Note that in the case of inorganic solids Cu–Kα radiation typically has an effective penetration depth of only a few tens μm.^50^

Figure 4c–e shows the 2D-XRD patterns and the calculated pole figures corresponding to the pseudomorphs dehydrated at increasing *T* as compared to those of the uncalcined gypsum control. The loss of diffraction spots corresponding to the parent gypsum single crystal, and the observation of clear Debye rings in the 2D-XRD patterns (which appeared independently of *T*) corresponding to the newly formed bassanite, suggest that its formation occurs without any clear structural control. These results are further confirmed by the lack of intensity maxima (i.e., complete absence of preferred orientation) in pole figures regardless of the selected Bragg reflection and calcination *T*.

Reconciling the significant disorientation of the product bassanite observed in 2D-XRD with the strongly preferred orientation of hemihydrate crystals and cracks evident in FESEM images is challenging. Since 2D-XRD data reflects the average orientation of the bulk material, signals from locally or surface-oriented crystals may be overshadowed. For this reason, it could be argued that the orientation of cracks along specific gypsum crystal planes/directions, and the observed orientation of the hemihydrate crystals (fishbone pattern) are only present at the surface of the pseudomorphs, whereas there is a lack of product phase orientation in their interior. Note that significant distortion can take place in the pseudomorph core due to pervasive shrinkage and cracking associated with the molar volume differences between gypsum and hemihydrate, creating significant porosity. It is therefore likely that the strain associated with the phase transition caused a general lack of clear orientation between the parent and product phases. In addition to this effect, one must consider the crystallization of randomly oriented α-hemihydrate within the gypsum pseudomorph (as opposed to the formation of oriented β-hemihydrate on the surface),^39^ confirmed here by OM analysis (Figure 1h,i) and consistent with previous studies.^39,42^ A similar process has been observed in polycrystalline gypsum rock.^7,49^ Trapped H_2_O creates high-*p*H_2_O “microenvironments”, resembling microhydrothermal chambers. In these water-rich microenvironments and at the corresponding experimental *T*, α-hemihydrate can form through nucleation and growth from solution rather than a solid-state process. This has been recently demonstrated by in situ synchrotron small- and wide-angle X-ray scattering (SAXS-WAXS) analysis of polycrystalline gypsum (Volterra alabaster) subjected to thermal dehydration in confinement where α-hemihydrate was observed to form via a dissolution–precipitation process due to the buildup of *p*H_2_O and subsequent condensation of liquid water.^7^ Note that hydrothermal (autoclave) conditions are required for α-hemihydrate formation.^51^ The buildup of *p*H_2_O (and subsequent condensation) within relatively large gypsum single crystals is also consistent with experimental results showing periodic fluctuations in dehydration rates measured during thermal decomposition of gypsum, which are associated with trapping and sudden release (after fracturing) of water vapor.^52^

In contrast, a topotactic solid-state reaction can occur in surface areas where the H_2_O release is facilitated, preventing the buildup of *p*H_2_O and favoring β-hemihydrate formation with preferred crystallographic orientations. Thus, it could be argued that maximizing the surface-to-volume ratio of gypsum crystals (e.g., by reducing crystal size) could favor topotactic replacement forming β-hemihydrate, whereas doing the opposite (using larger gypsum crystals) would enable through-solution precipitation of nonoriented α-hemihydrate during thermal dehydration.

To further disclose the role of crystal size in the thermal dehydration process, we analyzed the decomposition of gypsum and bassanite microcrystals in situ and in vacuo during TEM analyses following e-beam exposure. Figures 5 and 6 illustrate the effects of e-beam induced dehydration on gypsum microcrystals. The gypsum crystal in Figure 5a, oriented along the [010] zone axis (*I*2/*a* space group; Figure 5b), undergoes pseudomorphic transformation into an oriented aggregate of anhydrite nanoparticles (Figure 5c; *Amma* space group).^33^ The *post mortem* SAED pattern (Figure 5d; [010] zone axis of anhydrite) shows diffraction spots with an angular spread of ∼7–11°. We observed streaking of the Bragg reflection along the [10−1] (or equivalent [−101] ) direction (i.e., direction normal to the planes of primary cracking during thermal dehydration of gypsum), before e-beam focusing (Figure 5b), which could be partially responsible for the rapid transformation of gypsum into anhydrite. Streaking in SAED patterns is typically linked to diffuse scattering in an anisotropic, defective structure,^15^ and is most commonly observed in phases having planar defects (e.g., stacking faults) as well as dislocation arrays.^53^ Stawski et al.^54^ performed HRTEM-SAED analyses on laboratory-synthesized gypsum microcrystals and attributed the observed streaking and angular broadening to a strong mosaicity imprinted from the initial stages of gypsum crystallization, involving the oriented aggregation of nanometric subdomains. It is very likely that in the case of the Naica gypsum crystals studied here, the streaking has a similar origin. The possibility that the streaking is an artifact due to beam damage is ruled out here because the electron dose rate and total electron dose during the analysis of the gypsum microcrystals (Figure 5a,b) were very low (fluence: 49 e^–^/s Å^2^; total e-dose: 490 e^–^/Å^2^). Moreover, streaking and arcing of diffraction spots has been also observed during XRD analysis of gypsum single crystals, where beam damage can safely be ruled out.^55^

**Figure 5:**
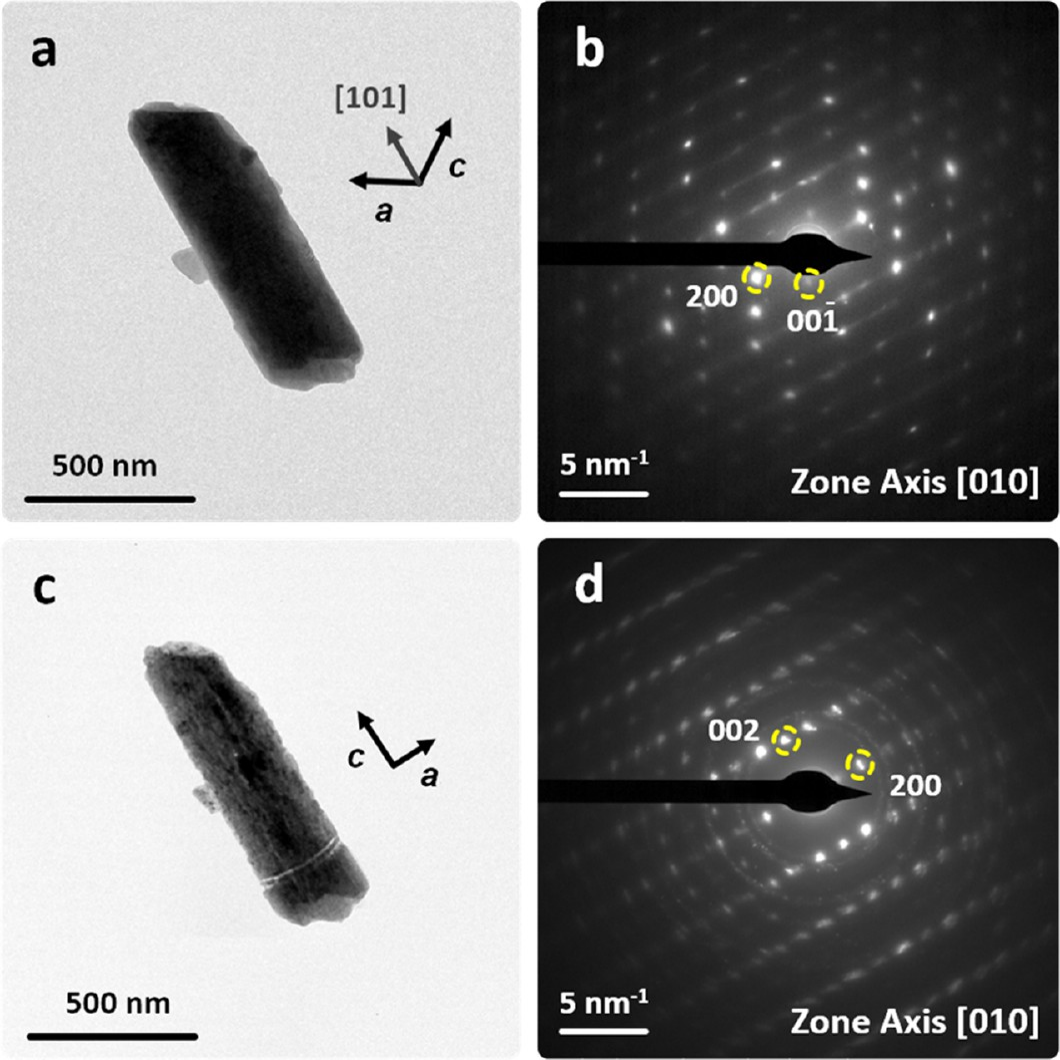
TEM images of a gypsum microcrystal undergoing in situ dehydration. (a,b) Micrograph and SAED pattern of the gypsum crystal in its initial state (e-dose rate: 53 e^–^/s Å^2^; total e-dose: 530 e^–^/Å^2^). (c,d) Micrograph and SAED pattern of the same crystal after dehydration induced by the e-beam revealing a clear [101]_Gyp_//[001]_Anhy_ orientation relationship, indicating that the transformation is topotactic (e-dose rate: 624 e^–^/s Å^2^, total e-dose: 5.6 × 10^4^ e^–^/Å^2^). The prolonged (90s) focused e-beam exposure causes partial CaSO_4_ decomposition into nonoriented CaO nanoparticles, evidenced by polycrystalline Debye rings in the SAED pattern of the pseudomorph (d).

**Figure 6:**
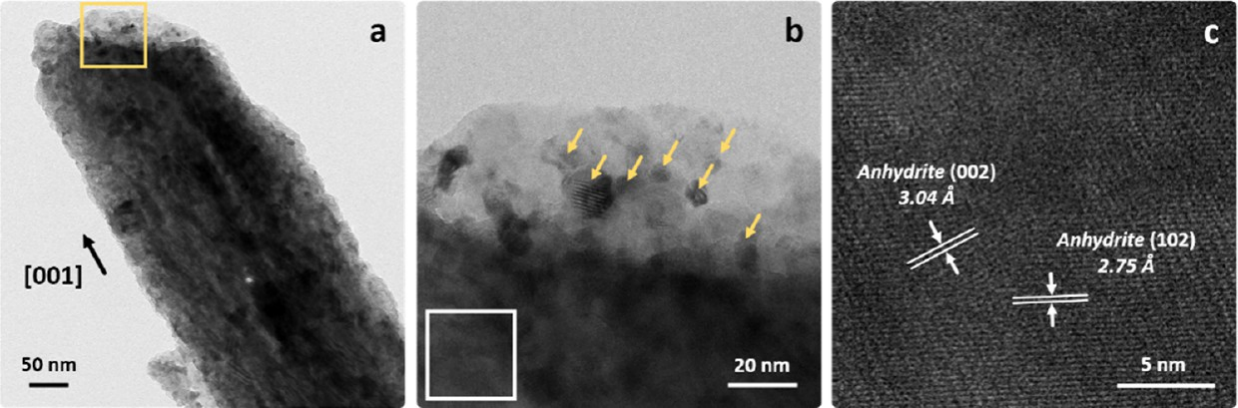
High-resolution TEM micrographs of the dehydrated gypsum pseudomorph showing the formation of oriented insoluble anhydrite aggregates (a,b), alongside nonoriented CaO nanoparticles (indicated by arrows in panel (b), corresponding to the squared area in (a)) (e-dose rate and total e-dose in (a) are 803, and 8030 e^–^/Å^2^, respectively). The HRTEM image in (c) shows lattice fringes of insoluble anhydrite (magnified squared area marked in (b)) (e-dose rate and total e-dose in (b,c) are 1.7× 10^4^, and 1.7 × 10^5^ e^–^/Å^2^, respectively).

The anisotropic gypsum-to-anhydrite transformation involves increased microstrain, potentially the cause of the slight orientation mismatch in the newly formed anhydrite nanoparticle aggregates. SAED analysis reveals the following reactant/product orientation relationships: [010] _Gyp_//[010] _Anhy_ and [101] _Gyp_//[001] _Anhy_, highlighting the preserved orientation of the Ca–SO_4_ chains in the parent and product phases (Figures 5b,d). Prolonged e-beam exposure resulted in the partial radiolytic decomposition of CaSO_4_, originating nonoriented aggregates of calcium oxide nanoparticles. This is evidenced by polycrystalline rings (111, 200, and 220 reflections) in the SAED pattern of the pseudomorph (Figure 5d) and the high-resolution TEM micrographs (Figure 6a,b). Formation of CaO after e-beam irradiation of anhydrite crystals in the TEM has been previously reported by Cao et al.^56^ The authors showed that the decomposition of CaSO_4_ into CaO upon e-beam exposure was not due to *T*-rise (which was calculated to be minimal), but rather to a radiolysis process. It should be underlined that here radiolysis should also be responsible for anhydrite formation upon gypsum dehydration as we used very similar 200 kV acceleration voltage, electron fluxes, and total dose as Cao et al.^56^ It could thus be argued that the mechanisms resulting in dehydration of gypsum in the TEM and in air upon heating are not comparable. Note, however, that even though the actual physical processes leading to dehydration of gypsum are not the same (radiolysis vs thermal decomposition), it has been demonstrated that the thermal decomposition of ionic solids follows the same mechanism and results in the same textural/structural features as the decomposition by e-beam irradiation in the TEM.^57^ The absence of bassanite or soluble anhydrite formation during in situ TEM measurements is likely due to the high energy from the e-beam and ultrahigh vacuum conditions, which should facilitate the rapid transition of gypsum to hemihydrate/soluble anhydrite and subsequently insoluble anhydrite, and the decomposition of the latter into nonoriented CaO nanoparticles (Figure 6a–c). It is important to underline, however, that the orientation relationships observed between gypsum and insoluble anhydrite would be similar to those between bassanite and soluble anhydrite as all these phases include Ca–SO_4_ chains along the direction of their *c*-axis. Altogether, these results indicate that crystal size plays a significant role in the dehydration of gypsum. In micrometer crystals, with a high surface to volume ratio, water is easily released, preventing condensation and secondary dissolution resulting in random nucleation of α-hemihydrate within the pseudomorph. In contrast, in larger millimeter-sized crystals, this process occurs primarily in the outermost layers or near the edges, where water release is not physically hindered, allowing for localized topotactic replacement. Comparable results were observed in the case of micrometric bassanite pseudomorphs formed after ex situ thermal dehydration of gypsum powder. The porous bassanite pseudomorphs diffracted as single crystals, confirming that the gypsum → bassanite transformation was structurally controlled and topotactic (Figure 7a,b). These results also confirm that the actual mechanism of micrometer-size gypsum dehydration resulting in bassanite with a clear topotactic relationship is not dependent on the energy source, that is, thermal (ex situ decomposition) or e-beam (in situ decomposition) energy.

**Figure 7:**
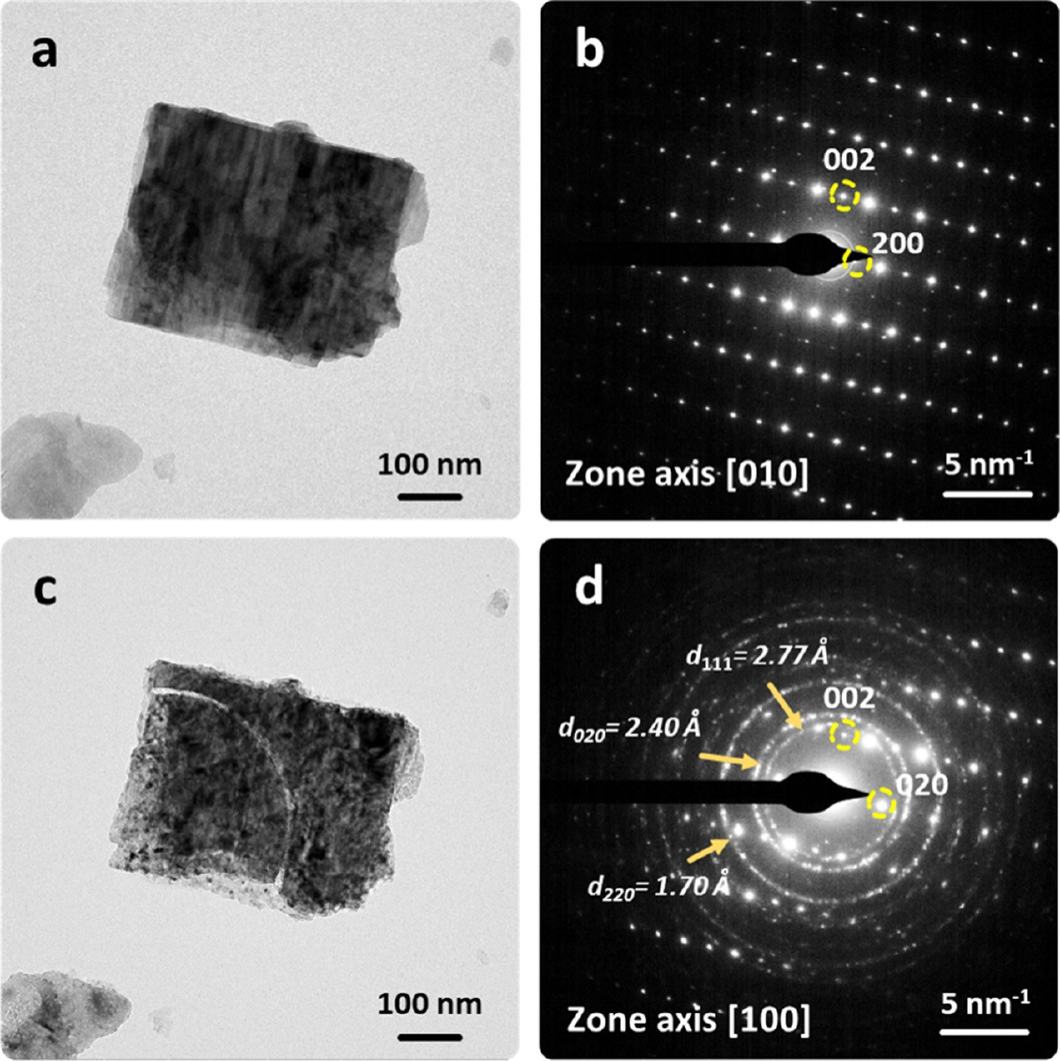
TEM images of a bassanite microcrystal (pseudomorph obtained by ex situ thermal dehydration of gypsum microcrystal) undergoing in situ dehydration. (a,b) Micrograph and SAED pattern of the bassanite crystal in its initial state (e-dose rate and total e-dose are 344, and 3.4 × 10^3^ e^–^/Å^2^, respectively). Note that it diffracts as a single crystal, demonstrating that there is a clear gypsum-to-bassanite topotactic transformation, because the parent phase was also a gypsum single crystal. (c,d) Micrograph and SAED pattern of the same crystal after transformation into insoluble anhydrite induced by the electron beam (e-dose rate and total e-dose are 2.9 × 10^3^ and 1.1 × 10^6^ e^–^/Å^2^, respectively). Note that low intensity *h*0*l* (where *l* = odd) diffraction spots were visible in the SAED pattern of the bassanite crystal, but not in the SAED pattern of the e-beam damaged crystal. This also evidence a clear [010]_Bass_//[100]_Anhy_ relationship indicating that this transformation is also topotactic. Polycrystalline rings correspond to CaO nanoparticles formed following the radiolitic decomposition reaction CaSO_4_ → CaO + SO_3_. The *d*_*hkl*_ spacings indicated in panel (d) correspond to CaO.

Further e-beam exposure of bassanite pseudomorphs (oriented along the [010] zone axis) led to the formation of a pseudomorph composed of insoluble anhydrite oriented along the [100] zone axis (Figure 7c,d). As noted, bassanite and anhydrite are practically isostructural, and the *d*-spacings corresponding to the *h*00, 0*k*0 and 00*l* reflections remain almost constant during the bassanite → anhydrite phase transition. However, the low intensity *h*0*l* (where *l* = odd) diffraction spots of bassanite and/or soluble anhydrite were absent in the SAED patterns of the beam-damaged pseudomorph, indicating that the newly formed solid phase was insoluble anhydrite, also diffracting as a single crystal. These results confirm that the dehydration reaction, whether involving gypsum or bassanite, is topotactic.

Prolonged electron beam exposure (up to 360 s) produced randomly oriented calcium oxide nanoparticle aggregates, as seen in the post-mortem SAED and FFT patterns, which showed no preferential orientation for the 111, 220, and 020 reflections. High-resolution TEM micrographs of the pseudomorph revealed nonoriented CaO nanoparticle lattice fringes (Figure 8b).

**Figure 8:**
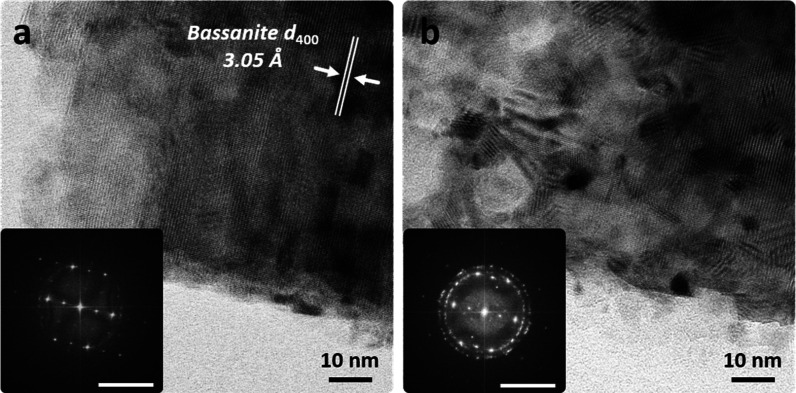
High resolution TEM micrographs and FFT patterns (insets) showing the dehydration of a bassanite single crystal (pseudomorph after ex situ thermal dehydration of gypsum) (a) into oriented insoluble anhydrite single crystal plus CaO aggregates (b) induced by the electron beam (e-dose rate and total e-dose in (a) are 7.5 × 10^3^, and 3.7 × 10^4^ e^–^/Å^2^, respectively; e-dose rate and total e-dose in (b) are 7.5 × 10^3^, and 1.8 × 10^6^ e^–^/Å^2^, respectively). Note that the same low intensity *h*0*l* (with *l* = odd) bassanite reflections (inset in a) are not visible in the FFT of the transformed crystal (inset in b). Non- oriented CaO nanoparticles, which are responsible for the ring pattern in the FFT, can be observed. Scale bars in FFTs correspond to 5 nm^–1^.

### 3.2 Rehydration of Bassanite

Millimeter-sized pseudomorphs subjected to rehydration under high *p*H_2_O conditions showed similar textural and morphological features as compared to the dehydrated state. In general, fishbone patterns and cracks observed on the surface of the pseudomorphs were preserved during hydration. However, detailed high magnification FESEM images revealed particles with a nanogranular texture, likely due to the regrowth of gypsum on the surface of bassanite microneedles present on the surface of the pseudomorphs (Figure 9a–c). This textural feature suggests that a highly supersaturated solution formed on the surface of bassanite particles upon dissolution (after deliquescence following exposure to the high RH atmosphere). Under these conditions high nucleation rates are favored, leading to the heterogeneous crystallization of nonoriented gypsum nanogranules. This is consistent with the overall disorientation observed in the 2D-XRD patterns and pole figures (Figure 9d). Furthermore, during the phase transition, particles show a globular aspect rather than an acicular morphology. This swelling and expansion effect likely is caused by the increase in molar volume associated with the hydration process (i.e., *V*_*m*‑Bass_ = 53.16 cm^3^ mol^–1^; *V*_*m*‑Gyp_ = 74.53 cm^3^ mol^–1^). The surface appearance of the hydrated pseudomorphs suggests a preferred orientation of the newly formed gypsum. However, only in the case of the partially dehydrated pseudomorphs obtained by calcination at 100 °C, the recorded 2D-XRD patterns after rehydration show diffuse arcs with intensity maxima, rather than full Debye rings (Figure 9d). This indicates that the newly formed gypsum grew in crystallographic register with the nondehydrated gypsum domains still present in the core of the low-*T* pseudomorph. The lack of topotaxy was confirmed by the 2D-XRD patterns of the pseudomorphs obtained at higher temperatures (i.e., 150 °C) where the gypsum → bassanite conversion was complete. This pattern shows continuous Debye rings for the newly formed gypsum, and no preferred orientation is observed in the pole figures. This supports the idea that bassanite rehydration occurs via a dissolution–precipitation mechanism, where the initial crystallographic features of gypsum are not retained. The preferred orientation observed in the low-*T* pseudomorph is thus attributed to a template effect (i.e., self-epitaxy). Self-epitaxy, facilitated by the presence of an unreacted gypsum core, likely accelerates the hydration reaction, which explains why adding gypsum seeds to plaster of Paris significantly speeds up its hydraulic setting.^58,59^

**Figure 9:**
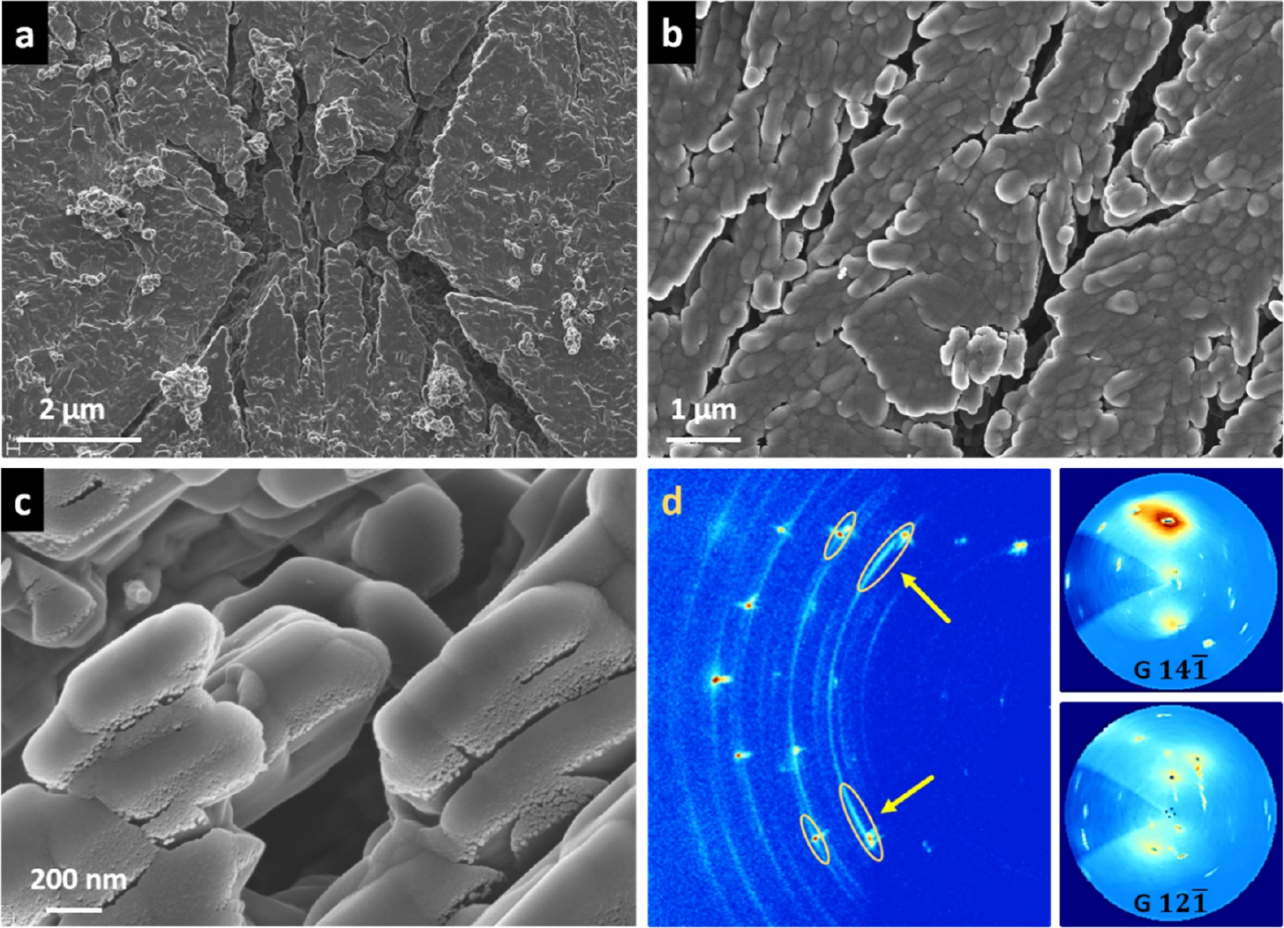
FESEM micrographs showing the textural aspect of rehydrated gypsum crystals under 100% RH for 12 months. The globular (a,b) and nanogranular (c) textures of the hydrated particles indicate dissolution of bassanite and reprecipitation of gypsum on the surface of pre-existent bassanite particles. These textural features contribute to the nonoriented fraction of the newly formed gypsum, as observed in the 2D-XRD patterns and pole figures of the rehydrated pseudomorphs. However, 2D-XRD patterns and pole figures corresponding to the rehydrated low-*T* pseudomorph (100 °C) (d) show nonhomogeneous Debye rings with intensity concentrated in arcs near the spots of gypsum single crystal, which are consistent with regrowth by self-epitaxy. induced by nondehydrated gypsum domains still present in the core of the pseudomorph.

Overall, these results align with the original model proposed by Le Chatelier^60^ and are supported by recent experimental findings showing that bassanite hydration can be explained by a dissolution/precipitation mechanism.^61^ They also point to a tight interface-coupled dissolution/precipitation process, as proposed by Putnis.^62^ According to Putnis’ model, high supersaturation with respect to the product phase occurs within the fluid in contact with the dissolving parent phase. In this interfacial fluid, geochemical conditions can differ significantly from those of the bulk solution, enabling fast, far-from-equilibrium reactions that favor the pseudomorphic transformation. The high supersaturation reached within this interfacial fluid is consistent with the nanogranular features of the newly formed gypsum (i.e., high nucleation density). Nevertheless, it remains unclear whether gypsum formation after bassanite involves the epitaxial growth of the former on specific crystal faces of the latter, as suggested by Jia et al.^63^ The absence of any preferred orientation between these two phases, determined here by 2D-XRD analysis, suggests, however, that no epitaxy exists. The latter is consistent with the detailed synchrotron study by La Bella et al.^61^ showing that there is no epitaxy during such a hydration reaction.

## 4 Conclusions

Our results show that the dehydration of gypsum to form bassanite and/or anhydrite, whether soluble or insoluble, is crystallographically controlled. However, microtextural variations upon phase transformation depend on three independent variables: temperature, *p*H_2_O and crystal size.

In the case of the thermal dehydration of relatively large (millimetric) gypsum single crystals (Figure 10a), different textural features were observed. On the surface, the intersection of three different sets of microfractures resulted in the formation of hourglass-shaped cracks. These cracks, here called type I, II and III, aligned parallel to the direction of the three main periodic bond chains (PBCs; [101], [100] and [001] respectively, according to the *I*2/*a* space group), as observed along [010]. Most notably, bassanite/anhydrite microneedles were observed arranging in “fishbone” patterns, with their long axis oriented at an angle of ∼35–45° with respect to the [101] direction. Even though product bassanite/anhydrite microneedles showed a regular spatial distribution pointing to a topotactic replacement, 2D-XRD analyses demonstrated that no orientation relationship existed between the parent and product phases, regardless of the calcination temperature. Further microtextural analysis showed random nucleation of nonoriented subsurface α-hemihydrate nanoparticles and microneedles, suggesting that *p*H_2_O build-up and subsequent water condensation occurred in the interior of large (millimeter-sized) single crystals. The two different pathways leading to the formation of hemihydrate/anhydrite contribute to the overall disorientation observed by 2D-XRD.

**Figure 10:**
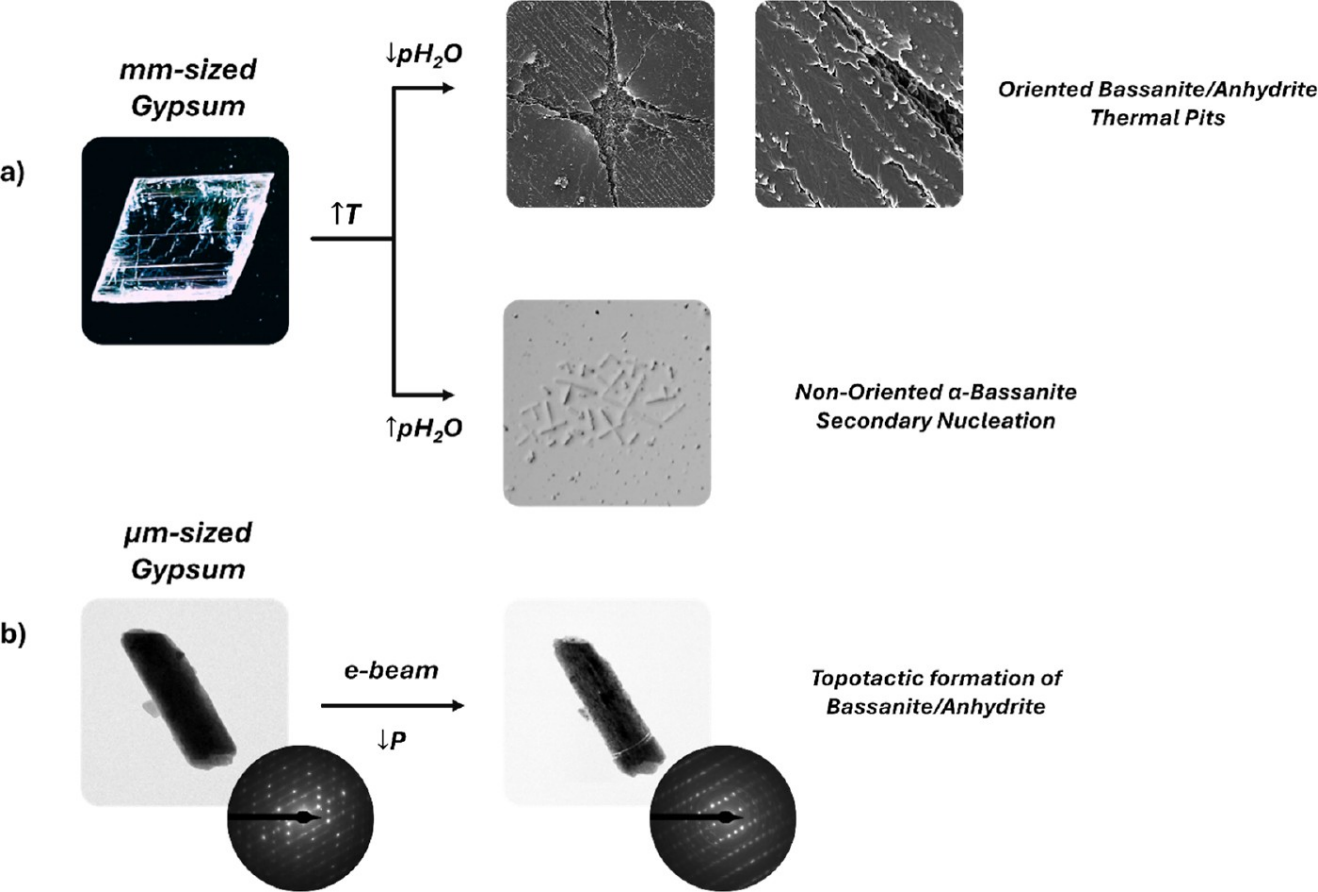
Schematic representations of gypsum dehydration pathways across the different experimental conditions tested: a) large mm-sized gypsum crystals and b) small micrometer-sized gypsum crystals.

In contrast, in situ TEM decomposition of gypsum microcrystals showed that this transformation is topotactic (Figure 10b). Sequential SAED patterns confirmed that parent gypsum and the resulting insoluble anhydrite pseudomorph diffracted as single crystals, and the [010]_Gyp_//[010]_Anhy_ and [101]_Gyp_//[001]_Anhy_ reactant/product orientation relationships were established. An analogous situation is found between bassanite and insoluble anhydrite, evidenced by the [010]_Bass_//[100]_Anhy_ (equivalent to [100]_Bass_//[010]_Anhy_) and [001]_Bass_//[001]_Anhy_ orientation relationships. Controlling gypsum crystal size during calcination determines whether topotactic replacement (producing highly porous, reactive β-hemihydrate or soluble anhydrite) or nontopotactic processes (yielding α/β-bassanite mixtures) prevail. These results may help explain the superior durability of ancient gypsum plasters prepared by traditional calcination of relatively large (cm to dm) pieces of gypsum rock, compared to modern industrial gypsum plasters typically prepared by calcination of crushed and grinded (to <150 μm) gypsum. Crushing should result in defect generation in the resulting gypsum microcrystals resulting in accelerated dehydration kinetics.^64^ However, the resulting product would be β-hemihydrate which generates set plasters of inferior mechanical properties as compared with those prepared using α-hemihydrate.^51^

Ultimately, the rehydration kinetics of bassanite and the textural features of the resulting set gypsum plaster would strongly depend on the crystal features and phase composition of the calcined gypsum and whether a topotactic replacement has prevailed or not. Highly reactive needle-like β-bassanite nanocrystals formed upon topotactic replacement would lead to a high nucleation density of small gypsum crystal, whereas bassanite (including α-hemihydrate) formed with little topotactic input, would be less reactive, forming more dense and durable structures.

Incomplete calcination retains untransformed gypsum domains that enable the epitaxial growth of gypsum, partially preserving the orientation of the original parent gypsum and accelerating hydration kinetics. Conversely, complete calcination results in random gypsum crystal formation through nonepitaxial nucleation during rehydration. Altogether, these results can have important implications in the understanding of the performance of gypsum plasters (ancient and modern) and in the optimization of their properties for applications spanning from built heritage conservation to sustainable modern construction and biomedicine.

## Supplementary Material



## Notes

The Supporting Information document includes data from SEM-EDS analyses of Naica gypsum single crystals, and photographs showing the disintegration of a gypsum single crystal dehydrated at 130 °C before and after the addition of liquid water (PDF)

## Abbreviations

2D-XRD: two-dimensional X-ray Diffraction
TEM: transmission electron microscopy
FESEM: field emission scanning electron microscopy
SAED: selected area electron diffraction.
