# Supplementary material for: Structural Insights into the Dehydration and Rehydration of Gypsum
Source: Cryst Growth Des. 2025 Apr 24;25(9):2830–42. doi: 10.1021/acs.cgd.4c01414 (PMC12150679; doi:10.1021/acs.cgd.4c01414)
Supplement: Supplementary file 1 [file cg4c01414_si_001.pdf]

Supplementary Information

STRUCTURAL INSIGHTS INTO THE DEHYDRATION AND RE-HYDRATION OF GYPSUM

Miguel Burgos-Ruiz[1], Kerstin Elert[2], Alejandro B. Rodriguez-Navarro[1], Encarnacion Ruiz-Agudo[1], and Carlos Rodriguez-Navarro[1]*

[1]Department of Mineralogy and Petrology, Faculty of Sciences, University of Granada, Avenida Fuentenueva S/N, Granada 18002, Spain.

[2]Escuela de Estudios Árabes, CSIC, Cuesta del Chapiz, Granada 18010, Spain

*Corresponding author e-mail: miguelburgos95@gmail.com

Table of Contents

Figure S1. SEM-EDS analysis of Naica gypsum single crystals.

Figure S2. Photographs of a gypsum single crystal dehydrated at 130 °C before and after the addition of liquid water.

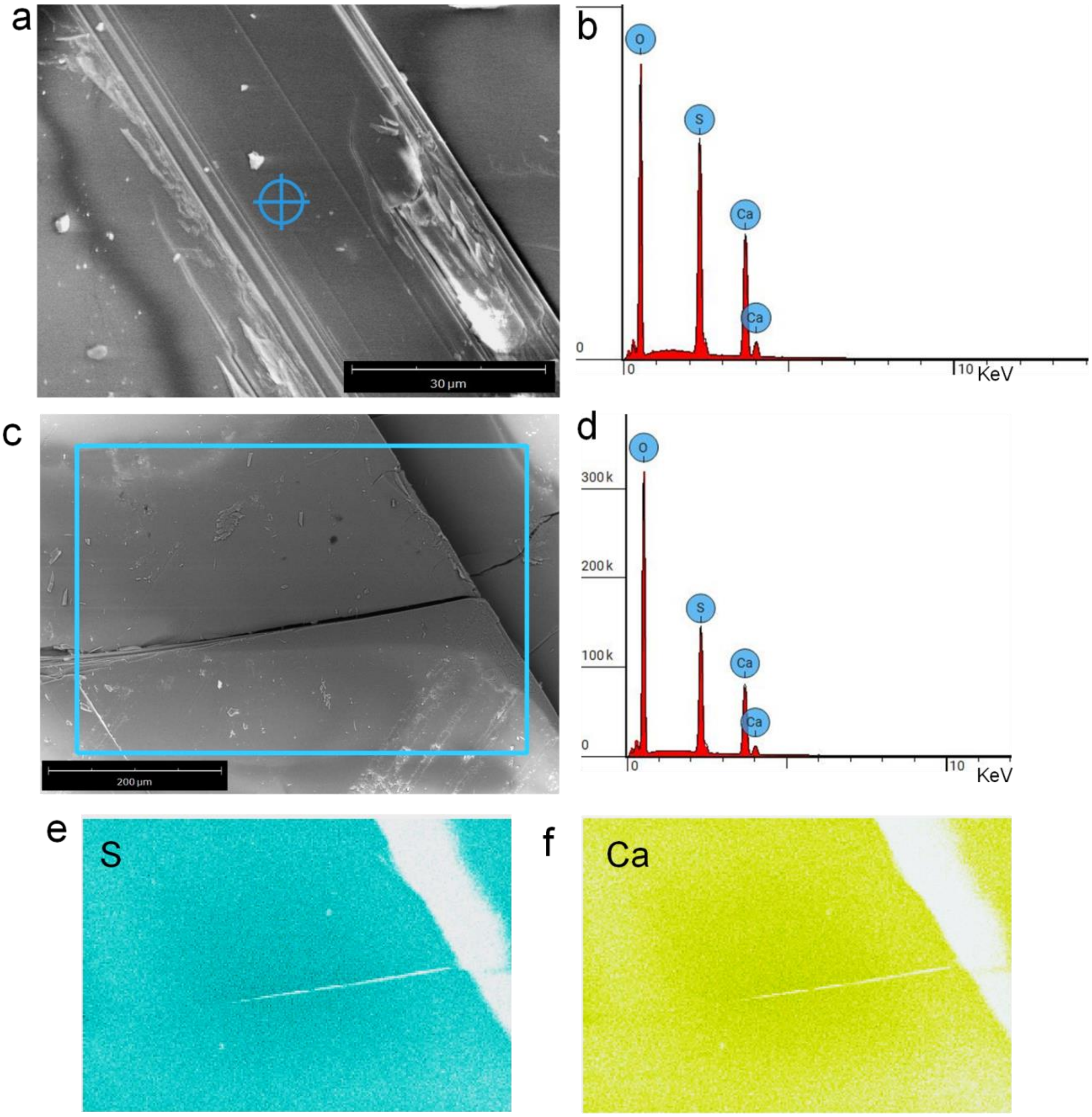

Figure S1. SEM-EDS analysis of Naica gypsum single crystals. a) SEM photomicrograph of the (010) cleavage face of gypsum; b) EDS spectrum of the point marked in the center of (a); c) SEM image of another gypsum crystal; d) Integrated EDS spectrum of the whole area marked by the blue rectangle in (b); S (e) and Ca (f) EDS maps of the area marked by the blue rectangle in (b).

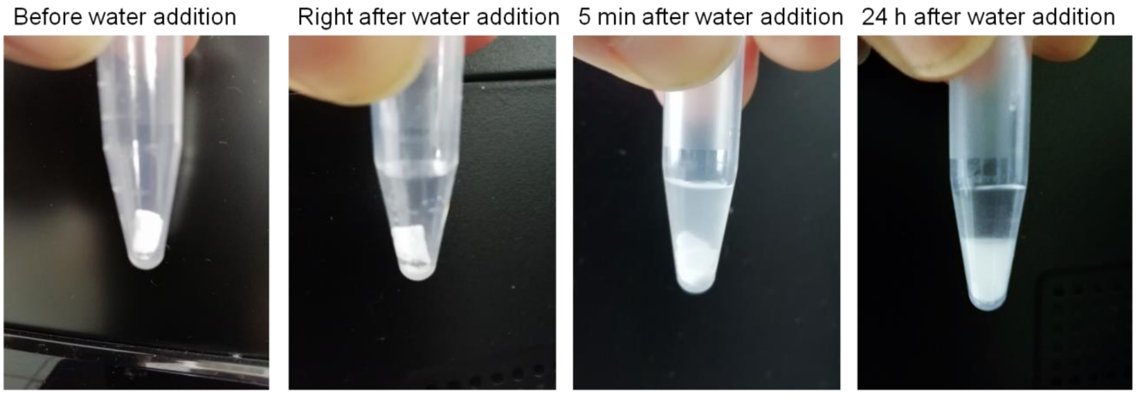

Figure S2. Photographs of a gypsum single crystal dehydrated at 130 °C before and after the addition of liquid water. Note its disintegration over time after water addition.
